# How to Sterilise Catgut

**Published:** 1904-07-09

**Authors:** 


### HOW TO STERILISE CATGUT.

THE value of catgut for purposes of ligature is not to be gainsaid, but hitherto there has always been a difficulty in sterilising it without diminishing its tensile strength or otherwise damaging it. Dr. Moschcowitz[1], after an extended trial, writes somewhat enthusiastically of catgut prepared by the iodine method as first suggested by Claudius. This method is as follows:—Catgut, just as it is bought from the dealer, is wound in a single layer on the spool, and tied at both ends to prevent unravelling. It is then immersed for at least eight days in a solution which contains one part of iodide of potassium and one part of iodine in 100 parts of distilled water, after which the material is ready for use. The ligatures may be kept in this solution for six months or longer, but as time goes on they lose some of their tensile strength, and it is therefore a good plan to prepare a fresh supply at the end of about six months. Dr. Moschcowitz remarks that at first he used to commence the sterilising process by removing the fat from the catgut with ether, but he has since found this to be unnecessary. The solution in which the ligatures are immersed should be kept in well-stoppered bottles to prevent deterioration through volatilisation of the iodine. When operating, Dr. Moschcowitz places the ligatures in sterile water, afterwards replacing the unused ones in the iodine solution for future use.

The writer claims for the method that it is efficient, easy of performance, and cheap, while the catgut prepared in this way is perfectly smooth, not swollen, very strong, yet soft and pliable and exceedingly convenient to use, as it does not kink or curl up like catgut which has been kept in alcohol. The knots stay tight and do not tend to untie or loosen, and there does not appear to be a tendency towards too early absorption of the material in the tissues.

[1] Med. Rec., May 14.

